# Laws of Health

**Published:** 1887-09

**Authors:** 


### LAWS OF HEALTH.

The human constitution has its laws, as definite and certain as those of astronomy. Cheerfulness and good will are of the first importance. Therefore, take the generous side and study benevolence and the welfare of others, as much for your own sake as theirs.

SUNLIGHT—is as essential to animal as vegetable life. Physicians say the number of patients cured in hospital rooms exposed to the rays of the sun, are four times as great as those confined in darkened rooms.

FRESH AIR—The air is the only agent which keeps the blood pure, and enables it to circulate and impart life-power to the system. It no less sustains life, by imparting this wonderful property to the blood, than by expelling the impurities or worn out matter which the veins have collected, and brought to the lungs for expulsion, and which, if left in the system but a very few moments, would cause death.

EXERCISE—is best if taken in some employment for an object. *Begin* and *end* slowly. It is well to carry our exercise to the point of fatigue if the system soon rallies from it; but for health, no greater fatigue should be incurred than a night's rest will remove. To sleep well and gain strength, the body must be fatigued.

FOOD—A free use of palatable fruit is essential. We must learn to distinguish between a real appetite and a mere superficial taste. The taste of sugar, for instance, may be agreeable, when there is no real need or appetite for it. Take few varieties of food at one meal. It is well, now and then, to omit by *turns* the use of every article of food—even bread, thus preventing the system from becoming tied to any injurious routine. It would not be amiss to make an occasional meal of some palatable fruit or vegetable in its season, when best relished.

WATER—An abundant supply and free use of pure soft water is essential to health. Water is the only fluid capable of circulating in all the tissues of the body, and penetrating its finest vessels without irritation or injury. No other liquid than water can dissolve the various articles of food taken into the stomach. It is water alone which forms the fluid portions of the blood, and thus serves to convey the nutriment to all parts of the body for its growth and replenishment. And it is water which takes up the decaying particles, and conveys them, by a most complicated and wonderful system of drainage from the body. When good soft water cannot otherwise be obtained, a small outlay for a cistern and filter will secure an abundant supply of pure rain water, equal to any.

BATHING—Much cold bathing exhausts vitality. Much warm bathing produces undue relaxation and sensitiveness· Hence, to secure the best results, avoid these extremes. The *temperature* of the water and surrounding air should be such as to allow a bath to be taken deliberately. With these conditions, and a moderately coarse towel, a yard in length, to supply the water, a very thorough bath may be taken. The towel bath affords excellent exercise for those engaged in sedentary employments.

An old New Yorker, who was brought up in hotels and restaurants, and knows all about eating, gave some points to a reporter of *The Sun* the other day, about the way for a man to make friends with his stomach. "There are two big mistakes that almost all persons make," said he. "One is that they don't eat the right things, and the other is that what they do eat they don't eat right. Dyspepsia and indigestion are killing more people than rum ten times over. If the stomach is right the head will be clear. No glutton or dyspeptic can stand up alongside of a man with a sound stomach and a clear head.

"When you got up this morning what did you do? Went right off to breakfast and filled yourself, with your nose in the papers and your mind wandering over the earth. You don't know what you ate, or how much or how long it took. For all the good it did you, you might as well have swallowed bacon and corn bread, or turkey and buckwheat cakes, or any other mixture that would take up space in your stomach. Then, while you ate, you gulped down ice water and coffee alternately, and when you got through you lit a cigar and went down town. glad you had done part of the work of the day.

"That's not breakfasting. It's loading up your stomach, and it's worse for you than if you hadn't eaten anything. Then you have a headache and feel bad, and grow fat, and wonder why it all is. It's because you don't pay as much attention to your stomach as you do to your office boy. Your stomach takes its revenge by making you wretched. To squelch it you pour a lot of liquor into it, and gulp some ice water on that with a cracker or pretzel and a bit of cheese. What sort of a mixture is that? Just imagine the cheese and rum and pretzel, and think that something inside of you has to get away with that. If you want to drink, drink and enjoy your drink. Don't down it and fling things at it when you've got it down. Take a glass of wine and enjoy it, but don't fling it into your stomach as you would your fist into somebody's eye. You stomach ought to be your friend, but if you go to pitching into it it'll show fight, and you may as well understand that it will get the best of it.

"When you get up in the morning take a big drink of water. Your system wants water first. An engine isn't first fired up and then some water let into the boiler. Clean your teeth, and let the water run from the spigot while you're doing it. Then drink a pint of it. Use common hydrant water ; no ice, no salt, no mineral water. Ordinary water is good enough for an ordinarily healthy man. Keep away from drugs and pills, and give your stomach a show.

"If you're in a hurry to read the papers, read them before breakfast. When you sit down to the breakfast table be happy ; you're going to do something pleasant. Breakfast isn't a penalty imposed on you or a task to be performed as soon as possible, but a pleasant, enjoyable occasion. Try and have somebody talk to you, and talk yourself. Laugh. Start off with fruit—some oranges, say. Then eat some fish and stale bread, or stale rolls or toast. If you want anything more, eat some meat. Take your time to it all. I stay at the table for an hour, and eat all the time. Don't eat much, but take your time to it. If you haven't time, eat less. The time you spend at breakfast will be saved over and over again during the day.

"If you've been up the night before, don't take a cocktail or ice water. Try some broth and some tripe if your stomach's pretty far gone. When a man's been off a little, his stomach is raw and inflamed. He doesn't want to start right off with more rum. Let him give his stomach a show. It'll pay him too. Coddle your stomach in the morning and it'll stand up for you at night. If you go pitching into it the first thing, it will have its revenge.

"Don't smoke in the morning. Don't drink in the morning. If you must smoke and you must drink, wait until your stomach is through with breakfast. Try this thing of starting off fair and square. You can drink more and smoke more in the evening, and it won't tell on it. A man's stomach is his friend, and if he'll only treat it kindly the first half of the day, it will show its appreciation and stick by him at night."

---

Glucose is a remarkable production. It is described in a recent French paper as follows: "Glucose—A product with which wine is manufactured without grapes, cider without apples, and confectionery without sugar."

